# Privatization of ophthalmology: unveiling disparities in the surgical arena

**DOI:** 10.1186/s13584-026-00756-1

**Published:** 2026-04-07

**Authors:** Asaf Israeli, Keren Hod, Rachel Shemesh, Tamara Wygnanski-Jaffe, Eedy Mezer

**Affiliations:** 1 Department of Ophthalmology, Vanderbilt Eye Institute, Vanderbilt University Medical Center, Nashville TN, USA; 2 Department of Nutrition Sciences, School of Health Sciences, Ariel University, Ariel, Israel; 3 Department of Academy and Research, Assuta Medical Center, Tel-Aviv, Israel; 4 Faculty of Medicine, Tel Aviv University, Tel Aviv, Israel; 5 Goldschleger Eye Institute, Tel Hashomer, Ramat Gan, Israel; 6 Bruce Rappaport Faculty of Medicine, Technion-Israel Institute of Technology, Haifa, Israel; 7 Department of Ophthalmology, Ruth Rappaport Children’s Hospital, Rambam Health Care Campus, Haifa, Israel; 8 Assuta Medical Centers, Haifa, Israel

**Keywords:** Disparities, Private, Public, Sex, Surgeries, Trends

## Abstract

**Purpose:**

To analyze the characteristics and trends of ophthalmic surgeries in public and private hospitals, as well as sex-disparities.

**Methods:**

A nationwide multicenter retrospective observational trend study encompassing surgeries in 9 different hospitals from 2017 to 2022. The retrieved data, which produced an anonymized database, was further divided into sub-specialties, before implementing descriptive and trend analyses.

**Results:**

A total of 97,325 surgeries were analyzed. Private hospitals had a higher female predominance (*p* < 0.001). A slight non-significant increase was noted in private hospitals over the years (0.9% annual increase, *p* = 0.07). Cataract and oculoplastic surgeries were the most common procedures in both domains. In private hospitals, male predominance was noted in cataract, glaucoma, corneal, retinal, and strabismus surgeries, whereas female predominance was noted in oculoplastic surgeries (*p* < 0.001 for each). In public hospitals, however, male predominance was only observed in glaucoma, corneal, and retinal surgeries, and female predominance was observed in cataract surgeries (*p* < 0.001 for each).

**Conclusion:**

Privatization seems to be an increasing trend in ophthalmology as well, and cream skimming is not as prevalent in this cohort. Sex differences were evident in both the public and private domains in different sub-types of eye surgery.

## Introduction

Privatization of healthcare is a growing phenomenon that sparks debate regarding the efficiency and benefit of the quality of care [1]. Patients requiring ophthalmologic surgery are often faced with the dilemma of choosing either public or private hospitals due to various reasons; however, sometimes no such choice exists due to geographical, social, or economic reasons; this occurs even in countries that offer universal healthcare [2]. 

This, some contend, affects equity in healthcare, as the right to care may be affected by the ability to pay for it. That, alongside equity-efficiency trade-offs, creates stark global debates among policymakers and different socioeconomic points of view. Core cultural, societal, financial as well as political views all play a major role in shaping a healthcare system [3–8]. 

The Israeli healthcare system specifically, which contains universal healthcare by law, has an unusual public-private framework – nonprofit health funds offer private insurance, public health funds own private hospitals, and public hospitals offer private services, which makes the situation all the more intricate [3]. Private healthcare in Israel is a rising trend, and many individuals choose to undergo surgeries in private hospitals rather than in public hospitals for various reasons (e.g., the better physical conditions, personal perceived autonomy, patient experience, and patient-provider communication) [9–11]. The term “Cream skimming” refers to “cherry picking” low-risk, profitable procedures, when considering care, over other justifications. This often means choosing younger, healthier patients [12–15]. Recent studies have reported “cream skimming” in ophthalmology; however, there are limited data regarding the magnitude and distribution of ophthalmologic surgeries within the public and private domains for all the various sub-specialties [16, 17]. 

Sex disparities were also reported in ophthalmology; males were more prone to retinal detachments and cataract complications, and more likely than females to undergo surgical corrections. Interestingly, a previous study in Israel showed no such disparity in strabismus surgeries [18–21]. 

The aim of the current nationwide study was to analyze the characteristics and trends of all types of ophthalmologic surgeries in both the public and private domains.

## Materials and methods

### Study participants, settings, and data collection

This retrospective observational trend analysis included 2 public tertiary university-affiliated hospitals – Rambam Health Care Campus (Haifa, Israel) and Sheba Medical Center (Ramat Gan, Israel); as well as Assuta Medical Centers’ Network of 7 private hospitals.

The data from the Electronic medical records (EMR) of all the ophthalmological surgical procedures from January 1st, 2017 to December 31st, 2022 were retrieved directly using MDClone^®^ software (MDClone Ltd., Beersheba, Israel), producing a large, anonymized database. The collected data included demographics (i.e., age and gender), the date of surgery, surgery duration, type of surgery, and surgery codes.

### Sub-specialty

The data collection included various procedural or surgical codes from all participating hospitals. These codes consisted of either ICD-9-CM Vol. 3 Procedure Codes or internal hospital codes. For procedures with ICD-9 codes, subspecialty grouping was performed using the eye-procedure ICD-9 categories (08–16) [22]. For internal codes, the subspecialty grouping was initially completed by hospital data specialists. Subsequently, Israeli and Mezer reviewed all classifications and manually revised them as needed. Ultimately, surgery types were grouped into the following six subspecialties: cataract, cornea, glaucoma, oculoplastic, retina, and strabismus.

### Statistical analysis

Statistical analyses were performed using the SPSS statistical package (Version 30, SSPS, Inc., Chicago, IL). The results are presented as the mean ± standard deviation (SD) for normally distributed continuous variables, as the median (interquartile range, IQR) for non-normally distributed continuous variables, and as frequencies for categorical variables. The one-sample Kolmogorov–Smirnov test results confirmed a normal distribution for the continuous variables. Comparisons between public and private domains and between genders were analyzed using Chi-square, Man-Whitney tests, or t-tests when appropriate. To address potential confounding factors, we performed additional multivariable logistic regression analyses to investigate the association between hospital type (private vs. public) and the subspecialty types of surgical procedures. Potential confounders identified in the univariate analyses were included in the regression models to control their influence and enhance the validity of the results. Six separate multivariable logistic regression models were constructed, each assessing the relationship between hospital type and a specific subspecialty surgical procedure. These models included: Model 1 (Oculoplastic), Model 2 (Cataract), Model 3 (Glaucoma), Model 4 (Cornea), Model 5 (Retina), and Model 6 (Strabismus). All models were adjusted for the same set of covariates. Adjusted odds ratios (ORs) with 95% confidence intervals (CIs) were calculated from each regression model. To evaluate trends over time, we conducted multiple regression analyses that incorporated relevant covariates. The models accounted for potential confounding factors, including age, sex, and specific subspecialty surgical type, to ensure that the results accurately reflected the underlying associations. The level of significance used for all analyses was two-tailed and set at *p* < 0.05.

## Results

The study included 97,325 surgeries performed during the study period [*n* = 72,138 (74.1%) private; *n* = 25,187 (25.9%) public]. Females comprised 56.1% of all patients (*n* = 53,975), and the median age was 69.4 years (IQR 60.3–75.7 years). Private compared to public hospitals had a significantly larger percentage of females (57.5%, *n* = 41,450 versus 52.2%, *n* = 13,139, respectively, *p* < 0.001), and the age was clinically similar (median 69.5 versus 68.9 years, respectively). Most surgical cases consisted of unilateral surgery (78.1%, *n* = 76,006) and there were significantly higher rates in public hospitals (91.6%, *n* = 21,597) compared with private hospitals (81.2%, *n* = 54,409, *p* < 0.001) (Table 1).

**Table 1:** Patient demographics and surgical characteristics in private and public hospitals

Variable	Private hospitals *N* = 72,138	Public hospitals *N* = 25,187	SMD	*P*	OR, 95% CI
**Patient demographics**					
Gender (female) n (%, 95% CI)	41,450 (57.5, 57.1–57.8)	13,139 (52.2, 51.5–52.8)	0.11	< 0.001***	1.24 (1.20–1.28)
Age (years), median (95% CI, IQR)	69.5 (65.8–66.1, 61.0-75.5)	68.9 (63.4–73.9, 57.6–76.3)	N.A.^1^	< 0.001***	1.01 (1.01–1.01)
**Surgery details**					
Surgery duration (minutes), mean ± SD (95% CI)	27.6 ± 14.6 (27.5–27.7)	27.1 ± 15.2 (26.9–27.3)	0.03	0.341	1.00 (1.00–1.00)
Total number of procedures, mean ± SD (95% CI)	1.0 ± 0.1 (1.0–1.0)	0.9 ± 0.1 (0.9-1.0)	1.0	0.875	5.56 (4.91–6.29)
**Procedure side**	N (%)	N (%)			
• Unilateral, n (%, 95% CI)	54,409 (81.2, 80.9–81.5)	21,597 (91.6, 91.3–92.0)	N.A.^2^	< 0.001***	2.54 (2.41–2.67)
• Bilateral, n (%, 95% CI)	12,605 (18.8, 18.5–19.1)	1976 (8.4, 8.0-8.7)	0.31	< 0.001***	0.39 (0.37–0.41)

CI, Confidence interval; IQR, Interquartile range; N.A., Not applicable; OR, Odds-ratio; SD, standard deviation; SMD, standardized mean differences

* Comparison is significant at the 0.05 level (2-tailed)

** Comparison is significant at the 0.01 level (2-tailed)

*** Comparison is significant at the 0.001 level (2-tailed)

^1^SMD not calculated for age, as the variable was non-normally distributed

^2^SMD is reported only for the bilateral category because unilateral and bilateral are complementary levels of the same binary variable

**Fig. 1:**
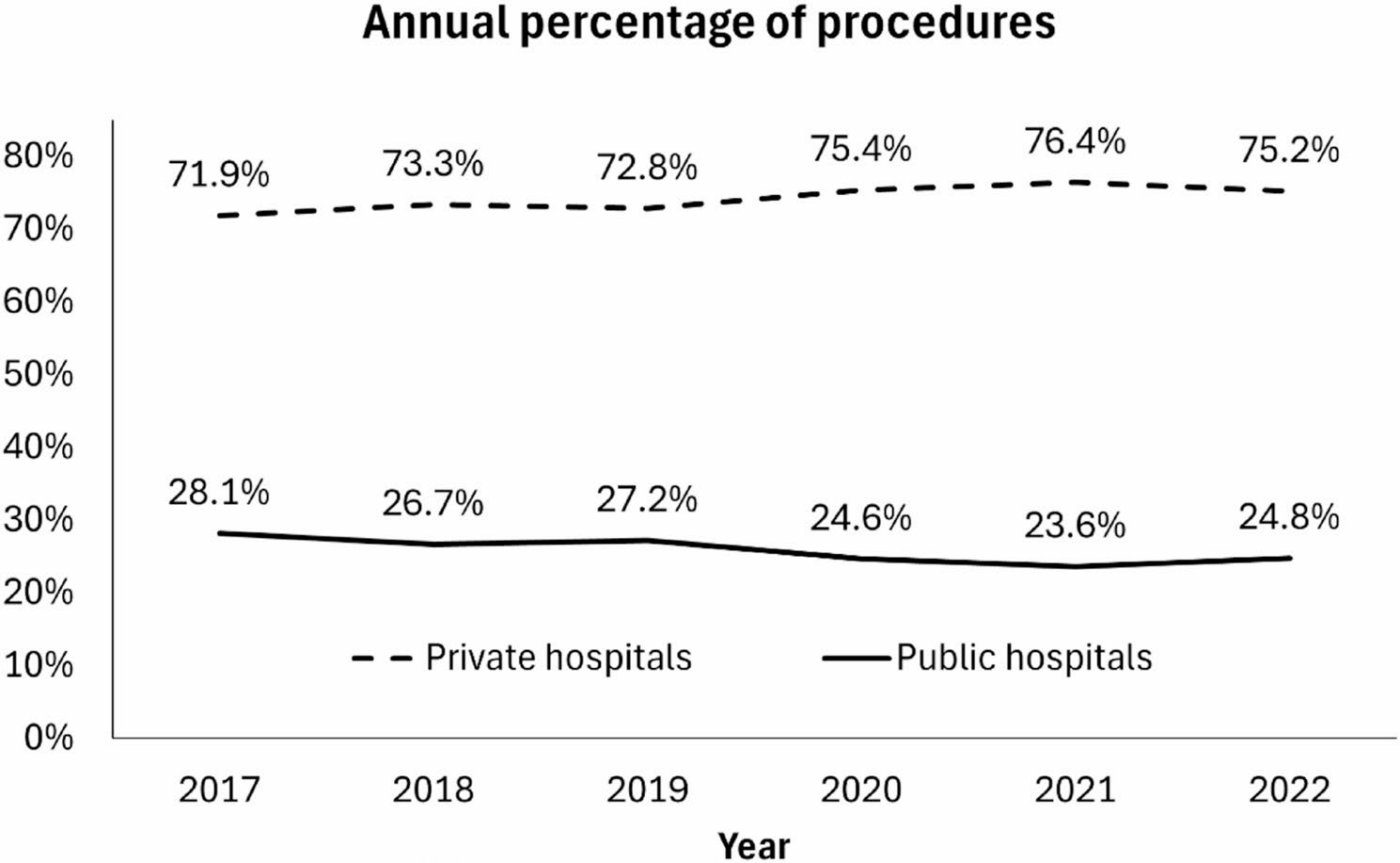
Annual percentage of surgeries in public and private hospitals from 2017–2022

**Fig. 2:**
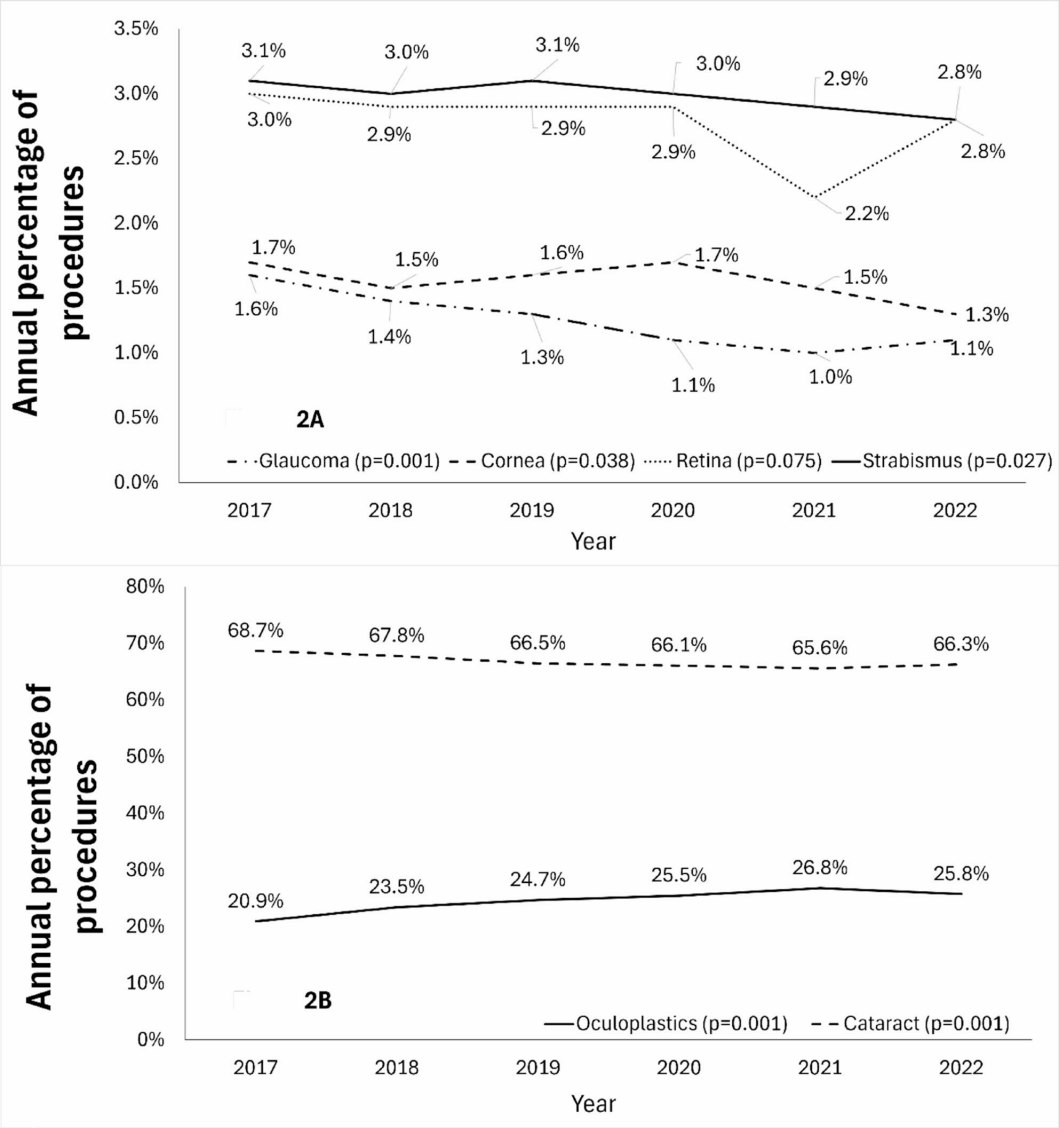
Annual percentage of surgeries according to the subspecialty of all surgical procedures (%) from 2017–2022

Overall, the percentage of surgeries conducted in a private setting increased from 2017 to 2022, with an average 0.9% annual increase (*p* = 0.07) and a reciprocal average annual decrease in the public sector (Fig. 1). In a multivariate model adjusting for age and gender, for the subspecialties, trends varied: oculoplastic procedures demonstrated a significant increase over time (*p* < 0.001), while cataract (*p* < 0.001), glaucoma (*p* < 0.001), cornea (*p* = 0.038), and strabismus procedures (*p* = 0.027) all showed significant declines to varying degrees (Fig. 2). However, the models exhibited very low R-squared values (0.000–0.002), indicating that the predictors accounted for are only a minimal portion of the variance in the year of the procedure. This suggests that other unmeasured factors likely contributed to the changes observed over time.

Cataract and oculoplastic surgeries were the first and second most common in both settings, respectively. Compared to public hospitals, cataract and strabismus surgeries were more commonly performed in private hospitals (69.6% vs. 59.0%; 3.3% vs. 2.1%, respectively) whereas all other types of surgeries were more frequently conducted in public hospitals compared to private hospitals (*p* < 0.001 for each, Table 2).

**Table 2:** Subspecialty types of surgical procedures conducted at private versus public hospitals

Subspecialty	All hospitals	Private hospitals: *n* (column %)	Public hospitals: *n* (column %)	OR, *P*-value
	Total *n* = 97,325 patients *n* (%, 95% CI)	Total *n* = 71,009 patients *n* (%, 95% CI)	Total *n* = 25,187 patients *n* (%, 95% CI)	
**Cataract**	65,076 (66.9%, 65.8–68.0)	50,216 (69.6%, 69.3–69.9)	14,860 (59.0%, 58.4–59.6)	1.67, < 0.001
**Oculoplastic**	23,809 (24.5%, 24.0–25.0)	17,653 (24.5%, 24.2–24.8)	6,156 (24.4%, 23.9–25.0)	0.95, < 0.001
**Strabismus**	2,897 (3.0%, 2.8–3.1)	2,369 (3.3%, 3.2–3.4)	528 (2.1%, 1.9–2.3)	1.61, < 0.001
**Retina**	2,715 (2.8%, 2.7-3.0)	1,076 (1.5%, 1.4–1.6)	1,639 (6.5%, 6.2–6.8)	0.22, < 0.001
**Cornea**	1,507 (1.5%, 1.0–2.0)	824 (1.1%, 1.1–1.2)	683 (2.7%, 2.5–2.9)	0.41, < 0.001
**Glaucoma**	1,223 (1.3%, 1.0-1.4)	457 (0.6%, 0.6–0.7)	766 (3.0%, 2.8–3.3)	0.21, < 0.001

*A total of 9 patients underwent 3 procedures, 813 patients underwent 2 procedures, and the rest (*n* = 95,574) underwent one procedure. Therefore, the total number of patients presented in the second row is lower than the sum of all procedures in each column

**Table 3:** Sex distribution of subspecialty types of surgical procedures conducted at private versus public hospitals

Subspecialty	Private hospitals: *n* (column %)		Public hospitals: *n* (column %)		OR, *P*-value^1;2^
	Male *n* = 30,173 *n* (%, 95% CI)	Female *n* = 40,836 *n* (%, 95% CI)	Male *n* = 12,048 *n* (%, 95% CI)	Female *n* = 13,139 *n* (%, 95% CI)	
**Cataract**	22,308 (72.7%, 71.4–74.0)	27,896 (67.3%, 67.0-67.6)	6,809 (56.5%, 56.0–57.0)	8,051 (61.3%, 60.0-61.6)	0.77, < 0.001; 1.22, < 0.001
**Oculoplastic**	6,166 (20.1%, 18.8–21.2)	11,487 (27.7%, 26.0-29.4)	2,978 (24.7%, 24.4–25.0)	3,178 (24.2%, 23.7–24.7)	1.52, < 0.001; 0.97, 0.328
**Strabismus**	1,180 (3.8%, 3.6–4.2)	1,189 (2.9%, 2.7–3.1)	255 (2.1%, 1.8–2.4)	273 (2.1%, 1.9–2.3)	0.74, < 0.001; 0.98, 0.830
**Retina**	526 (1.7%, 1.5–1.9)	549 (1.3%, 1.1–1.5)	904 (7.5%, 7.0–8.0)	735 (5.6%, 5.0-6.2)	0.77, < 0.001; 0.73, < 0.001
**Cornea**	480 (1.6%, 1.2-2.0)	343 (0.8%, 0.5–2.1)	416 (3.5%, 3.0–4.0)	267 (2.0%, 1.7–2.3)	0.53, < 0.001; 0.58, < 0.001
**Glaucoma**	240 (0.8%, 0.6–1.1)	217 (0.5%, 0.3–0.8)	423 (3.5%, 3.2–3.8)	343 (2.6%, 2.3–2.9)	0.66, < 0.001; 0.74, < 0.001

^1^ P-values and OR calculated between male and female within private hospitals

^2^ P-values and OR calculated between male and female within public hospitals

*A total of 9 patients underwent 3 procedures, 813 patients underwent 2 procedures, and the rest (*n* = 95,574) underwent one procedure. Therefore, the total number of patients presented in the second row is lower than the sum of all procedures in each column

**Fig. 3:**
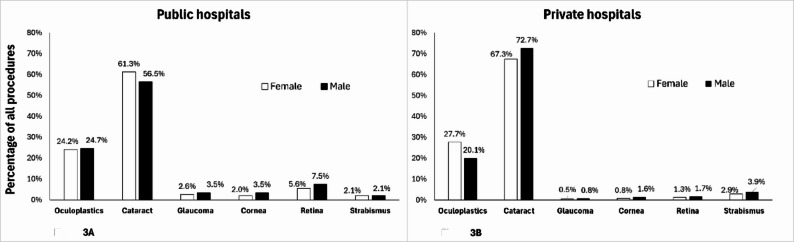
Sex disparities of the percentage of subspecialty surgeries in public and private hospitals

In private hospitals, male predominance was noted in all ophthalmologic surgery types, except for oculoplastic surgeries which was female predominant (*p* < 0.001 for each). In public hospitals, however, male predominance was only observed in glaucoma, corneal, and retinal surgeries (*p* < 0.001 for each); oculoplastic and strabismus surgeries were similar in both men and women (*p* = 0.328 and *p* = 0.830, respectively). Interestingly, a higher percentage of women than men underwent cataract surgery in public hospitals, in contrast to private hospitals (*p* < 0.001, Table 3; Fig. 3).

A multivariable logistic regression analyses exploring the association between hospital type (private vs. public) and subspecialty surgical procedures, adjusted for age and gender was employed. Gender was a significant factor across all models (Adj ORs: 1.22–1.26, *p* < 0.001), while age was significant in most models (Adj OR: 1.01, *p* < 0.001), except for cataract procedures, where it was not significant (*p* = 0.625). Among the procedure-specific models, oculoplastic procedures showed no significant association with hospital type (Adj OR: 1.02, *p* = 0.389). Cataract and strabismus procedures were significantly more likely to be performed in private hospitals (Adj OR: 1.68 and 2.95, respectively, *p* < 0.001), while glaucoma, cornea, and retina procedures were more commonly conducted in public hospitals (Adj OR: 0.20, 0.45, and 0.22, respectively, *p* < 0.001). These findings highlight clear differences in the distribution of subspecialty procedures between private and public hospitals, even after adjusting for key confounders, except for oculoplastic surgeries, that were no longer significant after correction for age and gender (Table 4).

**Table 4:** A multivariable logistic regression analysis examining the association between subspecialty types of surgical procedures and the type of hospital (private versus public), adjusted for age and gender

	Model 1			Model 2			Model 3			Model 4			Model 5			Model 6		
	Adj OR	95% CI	*p*	Adj OR	95% CI	*p*	Adj OR	95% CI	*p*	Adj OR	95% CI	*p*	Adj OR	95% CI	*p*	Adj OR	95% CI	*p*
**Control variables**																		
Gender	1.24	1.20–1.27	< 0.001	1.26	1.22–1.29	< 0.001	1.23	1.19–1.26	< 0.001	1.23	1.19–1.26	< 0.001	1.22	1.18–1.25	< 0.001	1.24	1.21–1.28	< 0.001
Age (years)	1.01	1.01–1.01	< 0.001	1.00	0.99-1.00	0.625	1.01	1.01–1.01	< 0.001	1.01	1.01–1.01	< 0.001	1.01	1.01–1.01	< 0.001	1.01	1.01–1.01	< 0.001
**Predictor variables**																		
Oculoplastic	1.02	0.98–1.05	0.389	--	--	--	--	--	--	--	--	--	--	--	--	--	--	--
Cataract	--	--	--	1.68	1.63–1.73	< 0.001	--	--	--	--	--	--	--	--	--	--	--	--
Glaucoma	--	--	--	--	--	--	0.20	0.181–0.229	< 0.001	--	--	--	--	--	--	--	--	--
Cornea	--	--	--	--	--	--	--	--	--	0.45	0.41–0.50	< 0.001	--	--	--	--	--	--
Retina	--	--	--	--	--	--	--	--	--	--	--	--	0.22	0.21–0.24	< 0.001	--	--	--
Strabismus	--	--	--	--	--	--	--	--	--	--	--	--	--	--	--	2.95	2.65–3.27	< 0.001

Adj OR-adjusted odds ratio, CI-confidence interval

Each model assesses the relationship between hospital type and a specific subspecialty surgical procedure. These models included: Model 1 (Oculoplastic), Model 2 (Cataract), Model 3 (Glaucoma), Model 4 (Cornea), Model 5 (Retina), and Model 6 (Strabismus)

Separate models were fitted as each subspecialty represents an independent clinical comparison of interest

## Discussion

Dual practice is a common and controversial phenomenon in today’s medicine and has significant bearing on public resources. While financial considerations are paramount, they are not the sole motivators, and this too varies significantly between different fields [23–25]. This multicenter study assessed surgical trends in public and private settings in Israel.

The popularity of private ophthalmologic surgical procedures has been gradually growing during the study period, corresponding to similar trends worldwide, in spite of the lack of evidence of healthcare benefits regarding the quality of care [1]. Notably, the dissimilarities do not always stem from patient preferences, since in some instances there is a need for urgent care (e.g., retinal detachment repair, recent onset strabismus, combined therapy for thyroid eye disease, trauma, tumors, and cerebrovascular accidents) [17, 26, 27].

Overall, the most prevalent ophthalmologic surgeries performed in our cohort were cataract and oculoplastic surgeries. Cataract surgery is more commonly performed in private institutions due to its brevity, enabling multiple daily procedures and financial gain; there are often backlogs in surgical waiting times. Cataracts remain a significant cause of visual impairment and blindness in Israel, despite its universal healthcare system [28, 29]. Esthetics and older life expectancy also play a role in the increasing demand for oculoplastic procedures, such as eyelid surgeries, which are brief extraocular procedures not requiring advanced machinery, thus contributing to lower costs and higher revenues.

Despite narrower age differences, age was not clinically different among patients undergoing surgeries in private compared to public institutions, in contrast to the “cream-skimming” phenomenon previously reported in Denmark or in strabismus surgeries in Israel [17, 30]. Possible explanations could be the lack of a “cherry picking” tendency in other sub-specialties, or merely a reflection of a better socioeconomic status as people grow older, contrary to patients undergoing strabismus surgeries, which mostly consist of pediatric patients.

In private hospitals, cataract, glaucoma, corneal, retinal, and strabismus surgeries were characterized by male predominance, however, oculoplastic surgeries were predominantly performed in females. This cannot merely be explained by the prevalence of common oculoplastic diagnoses; [31] it may also be related to increased psychosocial disparities between the sexes as well as esthetic concerns [32, 33]. In public hospitals, male predominance was noted in glaucoma, corneal, and retinal surgeries, all of which are usually intraocular, longer, and more complex procedures compared with cataract and oculoplastic surgery. Conflicting evidence exists in different parts of the world with regard to a stronger inclination to operate on males rather than females. The results in our cohort are similar to reports indicating that retinal surgeries are more common in men [13]. However, regarding cataract sex disparity, the higher surgical rates reported in females was similar only to our findings in public institutions [34, 35]. The higher percentage of men undergoing oculoplastic, glaucoma, corneal, and retinal surgeries in public hospitals may have resulted in a lower percentage of cataract surgeries. Nonetheless, it was still the highest among all subspecialties. It is worth mentioning that although previous studies reported that glaucoma is more prevalent among females, in Israel the incidence of blindness from glaucoma is higher in males [36]. 

Adjusting age and gender in multivariate analysis, oculoplastic surgeries showed no association with hospital type. Cataract and strabismus surgeries were more commonly performed in private hospitals, whereas glaucoma, corneal, and retinal procedures were more frequently conducted in public hospitals. This is likely due to the urgent nature of certain procedures in these fields, compared to cataract and strabismus which are often performed electively. Notably, a differential gender-specific analysis reveals disparities between different hospital settings. Sex-differences persisted even after adjusting for age and subspecialty. Sex-subspecialty interaction effects were considered but were not supported by the data.

This study has several limitations: First, its retrospective nature. Second, analyzing “big-data” of general surgical procedures according to subspecialties limits our ability to explore specific surgery types and individuals’ perioperative information, as well as surgical urgency. This, alongside additional possible relevant parameters (e.g., socioeconomic status) could also potentially affect the results. However, it improves statistical accuracy and generalizability. Third, although this study was comprehensive, nationwide, and multicenter, the cohort only included several hospitals in a single country, However, previous studies showed Israel had many similarities to other western countries [28, 29, 37]. 

Lastly, it should be noted that the patterns in this study are descriptive, and the R-squared values in our models indicate that a large portion of the variability is not explained by the data that was available to us (e.g., socioeconomic status and insurance characteristics, which are likely to affect private sector utilization and confound the reported associations - particularly in bilateral surgery rates, cataract utilization as well as cosmetic procedures). This could indicate that many other individual- and system based-parameters likely contribute meaningfully to the observed patterns and trends – including but not limited to ethnicity, supplemental insurance, waiting times, facility availability, and reimbursement incentives, to name a few. Future studies taking those and other clinical, social, and behavioral parameters into account could shed more light on the effect size of these factors.

## Conclusions

Privatization seems to be a slowly increasing trend in ophthalmology as well, but cream skimming only seems to be prevalent in certain types of surgeries (e.g., strabismus). Sex disparity was evident in different sub-types of eye surgery in both the public and private domains. Further research in different geographical areas and healthcare systems as well as exploring specific surgeries and financial incentives may be needed to better understand the underlying rationale.

## Data Availability

The data that support the findings of this study are available from the included hospitals, but restrictions apply to the availability of these data, which were used under license for the current study, and so are not publicly available. Data are, however, available from the authors upon reasonable request and with permission of the hospitals included.
